# Experimental temporal quantum steering

**DOI:** 10.1038/srep38076

**Published:** 2016-11-30

**Authors:** Karol Bartkiewicz, Antonín Černoch, Karel Lemr, Adam Miranowicz, Franco Nori

**Affiliations:** 1 Faculty of Physics, Adam Mickiewicz University, PL-61-614 Poznań, Poland; 2 RCPTM, Joint Laboratory of Optics of Palacký University and Institute of Physics of the Czech Academy of Sciences, 17. listopadu 12, 772 07 Olomouc, Czech Republic; 3 Institute of Physics of the Czech Academy of Sciences, Joint Laboratory of Optics of Palacký University and Institute of Physics of the Czech Academy of Sciences, 17. listopadu 50A, 77207 Olomouc, Czech Republic; 4 CEMS, RIKEN, 351-0198 Wako-shi, Japan; 5 Department of Physics, The University of Michigan, Ann Arbor, MI 48109-1040, USA

## Abstract

Temporal steering is a form of temporal correlation between the initial and final state of a quantum system. It is a temporal analogue of the famous Einstein-Podolsky-Rosen (spatial) steering. We demonstrate, by measuring the photon polarization, that temporal steering allows two parties to verify if they have been interacting with the same particle, even if they have no information about what happened with the particle in between the measurements. This is the first experimental study of temporal steering. We also performed experimental tests, based on the violation of temporal steering inequalities, of the security of two quantum key distribution protocols against individual attacks. Thus, these results can lead to applications for secure quantum communications and quantum engineering.

Einstein-Podolsky-Rosen (EPR) steering refers to strong nonclassical nonlocal bipartite correlations. It was first described by Schrödinger^1^ as a generalization of the EPR paradox^2^. The recent celebration of the 80 years of steering and the EPR paradox^3^ showed that our understanding of this phenomenon is now much deeper, but still very limited. Steering differs from quantum entanglement^4^ and Bell nonlocality^5^,^6^,^7^, as not every entangled state manifests steering and not every state that manifests steering violates Bell’s inequality^8^. In other words, steerable states are a subset of entangled states and a superset of Bell nonlocal states. Analogously to Bell nonlocality, steering can be detected independently of other nonclassical correlations by simple inequalities^8^,^9^,^10^ that can include as little as two measurements with two outcomes for Alice and a set of four possible states for Bob^9^. Such inequalities were tested in several experiments^10^,^11^,^12^,^13^,^14^,^15^,^16^,^17^, including a recent loophole-free experiment^18^. Steering can be interpreted as a correlation between two systems (measuring devices), where only one of them is trusted. This property shows an operational meaning of steering and indicates its potential applications in quantum cryptography and quantum communication, e.g., for entanglement distribution^8^,^19^. Steering-based protocols can provide secure communications even when only one party trusts its devices. Such protocols are easier to implement than completely-device-independent protocols^20^, but are more secure than standard protocols requiring mutual trust between the communicating parties.

Temporal steering^21^ (TS), analogously to EPR steering, is observed when Alice can steer Bob’s state into one of two orthogonal states by properly choosing her measured observable. Despite this similarity, the implications of these temporal and spatial phenomena are fundamentally different. To detect TS^21^, Alice and Bob perform consecutive measurements (using a random sequence of mutually-unbiased bases known only to them) on the same system to test temporal correlations between its initial and final states. Breaking the temporal steering inequality, given in ref. 21, implies that no unauthorised party can gather full information about the final quantum state. In other words, there was no quantum collapse and the observed correlations are stronger than any correlations between the initial state and its classical copy prepared by measuring and resending the initial state. Such strong temporal correlations must have a quantum origin. Their stronger form asserts that no third party can gather more information about the original state than Bob. In this case, Alice and Bob witness temporal correlations of a unique strength, which prove that they interact with the original quantum system and not with one of its quantum copies. This unique relation between the past and the future is referred to here as monogamous quantum causality. Here, Alice mostly steers the future state of Bob and no other system can be steered with the same strength. This regime is especially interesting for quantum cryptography, because it allows performing secure quantum key distribution protocols over a quantum channel, which is not fully characterised (trusted).

In contrast with EPR steering^10^,^11^,^12^,^13^,^14^,^15^,^16^,^17^,^18^,^22^,^23^,^24^,^25^,^26^,^27^,^28^,^29^,^30^, TS has not yet been investigated experimentally. This article reports, to our knowledge, the first experimental demonstration of TS. We verify, in a quantum linear-optical experiment, the relation between TS and two quantum key distribution (QKD) protocols based on mutually unbiased bases (MUB). Specifically, we apply temporal steering for experimental testing the security of the Bennett-Brassard 1984 protocol (BB84)^31^ and the six-state 1998 protocol by Bruss (B98)^32^ against individual attacks. As discussed theoretically in ref. 33, the unconditional security of these protocols^34^,^35^,^36^ (even against individual attacks) implies the existence of a kind of monogamous temporal correlations. The first experimental test of this temporal steering monogamy is reported here.

TS is understood as the ability of Alice to prepare a quantum object in a quantum state that after travelling, for a period of time through a damping channel, to Bob will manifest strong temporal correlations between its initial and final states. These correlations tell us how strong is the influence of Alice’s choice of observable on Bob’s results. The channel can erase partially or completely Alice’s influence. This decoherence process will take some time. Thus, TS is an appropriate name for this effect. It was shown^21^ that these temporal correlations are related to the one-way security bound in BB84. Therefore, this new kind of steering, similarly as the standard EPR steering, can be responsible for secure (one-way) quantum communications. However, ref. 21 did not explain the origins of this relation between TS and QKD. In a certain sense, this TS is a kind of one-way (or asymmetric) (temporal) steering because of the time arrow. For spatial steering, one could consider one-way (spatial) steering as well as two-way (spatial) steering, where the roles of Alice and Bob are interchanged. For TS this can be done only for a unitary (reversible) evolution of a given steered system. Steering is, by definition, asymmetric, as corresponding to one-side device-independent entanglement detection. Thus, in steering one assumes that only one side is performing faithful measurements. This is in contrast with entanglement, where both parties are trusted, as well as Bell’s nonlocality, where both parties are untrusted.

## Temporal steering

**Figure 1:**
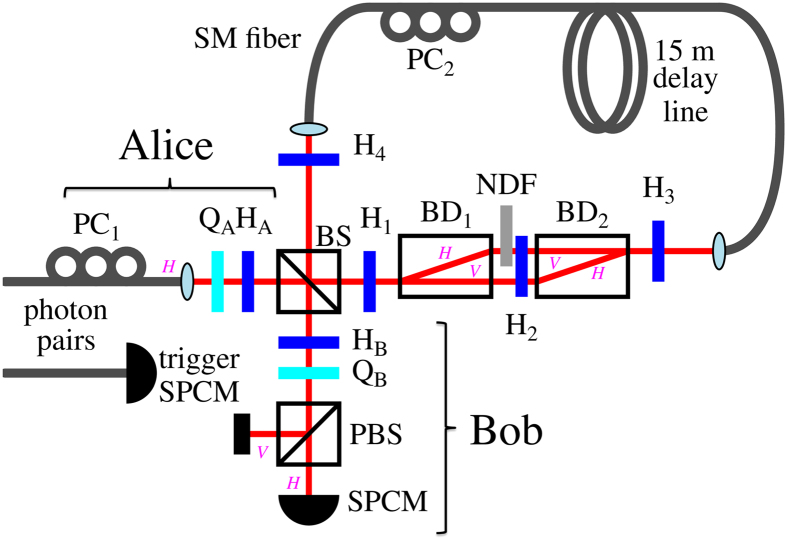
Experimental setup for demonstrating temporal steering. Here we use abbreviations: BS for beam splitter, PBS for polarising BS, NDF for neutral density filter of tunable transmittance *τ*, BD_*n*_ for beam dividers, PC_*n*_ for the polarisation controllers compensating polarisation rotation in the fibers, Q_*n*_ for quarter-wave plates, H_*n*_ for half-wave plates, SPCM for single-photon counting module, SM for the 15 m long single-mode optical fiber which forms a delay line of *t* = 80 ns (this is larger than the 50 ns long dead time of the SPCM). Alice prepares the initial state by rotating a *H*-polarised photon with Q_A_ and H_A_ plates. Bob performs state analysis by setting his measurement basis with H_B_ and Q_B_, and detecting the incoming photon in one of the orthogonal polarisation states.

In our experiment, Alice with probability *P*(*a*|*A*_*i*_) prepares qubits by rotating |*H*〉 (a horizontally-polarised photon) to one of the six eigenstates of the Pauli operators. This is done by the consecutive use of half- (H) and quarter-wave (Q) plates as shown in Fig. 1. To implement BB84, Alice sends eigenstates of only the *σ*_1_ and *σ*_2_ operators. She implements B98 by including also *σ*_3_. This method of state preparation is equivalent to performing a projective nondestructive measurement *A*_*i*_ by separating states of *a* = +1 and *a* = −1 with a polarising beam splitter (an equivalent of the Stern-Gerlach experiment^37^), and detecting their presence in one of its paths. A photon with probability *P*(+1|*A*_*i*_) chooses the path designated for *a* = +1 and, with probability *P*(−1|*A*_*i*_), the path for *a* = −1. However, the latter approach would be much more difficult to implement because it would require a nondemolition photon-presence detection (see, e.g., ref. 38). In the former approach, we assume that the state preparation governed by the probability distribution *P*(*a*|*A*) is equivalent to Alice’s nondestructive equiprobable measurements of *A*_*i*_ = *σ*_*i*_ for *i* = 1, 2, 3, where the outcomes of the measurement *A*_*i*_ are *a* = ±1 and appear with the probability *P*(*a*|*A*_*i*_) = 1/2. The final measurement does not have to be nondestructive, because this is the final step of the measurement. This is sufficient to check if the channel can partially or completely erase Alice’s influence on the state detected by Bob, hence to demonstrate TS without applying a quantum nondemolition measurement.

A nontrivial case of TS requires a nonunitary dynamics. We implement such evolution with two channels labelled by *λ*. To show this evolution, we analyze the TS parameters 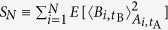 corresponding to the left-hand-side of the TS inequality of Chen *et al*.^21^ (see also ref. 33) and a measure of TS, i.e., the so-called TS weight *w*_*t*,*N*_, as defined in refs 33 and 39. The TS inequality is satisfied for all classical states and it reads


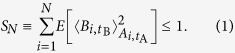


The technical definition of *w*_*t*,*N*_ is rather complex, thus we present it in the Methods. However, this quantity measures the amount of the genuine temporal steering correlations between Alice and Bob, maximised over the possible Bob’s measurements. The TS parameter *S*_*N*_ depends on the number *N* = 2, 3 of unbiased measurements *B*_*i*_ ≡ *σ*_*i*_ performed by Bob. This corresponds to a sum over the measurements of the expectation values 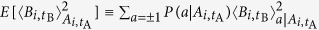, where Bob’s outcomes are related to the state projection performed by Alice, as 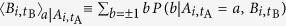. The parameter *N* represents the number of the MUB used by Bob to analyze the received qubit. For only one channel (corresponding to a simple unitary evolution of an isolated photon), the TS parameters and the TS weight would not exhibit an interesting behaviour beyond simple oscillations. In the extreme case, when the output state of a channel is always the same, independent of the input state, Alice’s influence on the state detected by Bob is completely erased. However, for typical imperfect channels, this Alice’s influence is only partially lost and, in the context of QKD, this loss of information can be attributed to eavesdropping.

For both BB84 and B98 there exists a minimal value of the average quantum bit error rate (QBER) *r*_*N*_ in the raw key for which the respective protocol is no longer secure. For individual qubit attacks these values are 
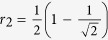
 for BB84 (*N* = 2) and 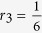 for B98 (*N* = 3). These values correspond to the minimal amount of noise introduced by an eavesdropper equipped with a quantum cloning machine optimised to copy the states prepared by Alice in a relevant protocol. The security of these QKD protocols is naturally related to optimal quantum cloning and it was studied in various works (see, e.g., refs 36, 40, 41, 42, 43 and references therein). It was recently shown that the TS parameter *S*_*N*_ depends on the average QBER *r*_*N*_ or the average fidelity *F*_*N*_ = 1 − *r*_*N*_ of the states received by Bob^33^. This was done by expressing the steering parameter *S*_*N*_ in terms of the fidelity *F*_*i*,*a*_ of the particular states prepared in a QKD protocol by Alice with respect to the states measured by Bob, i.e., 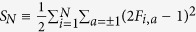. The derived relation between temporal steering and security of QKD^33^ *S*_*N*_ > *N*(1 − 2*r*_*N*_)^2^ asserts that QBER_*N*_ < *r*_*N*_. Thus, the minimal violation of this security condition indicates the maximal values of *S*_*N*_ = *N*(1 − 2*r*_*N*_)^2^ for which the relevant QKD protocols are insecure.

In our experiment, *λ* can have two values 0 and 1. For *λ* = 0, a photon of polarisation *V* is erased, with probability *p*_0_ = 1 − *τ*, with a filter of transmittance *τ*. For *λ* = 1, with probability *p*_1_ = 1 − *p*_0_, the photon is passed to Bob in the state *R*(*θ*)|*a*|*A*〉. The polarisation is rotated by an angle *θ*, i.e., it is transformed by the operator 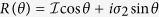, where 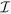 is the two-dimensional identity operator. Each time a photon is erased by the filter, Bob counts one photon received in state *R*(*θ*)|*H*〉. We can set the values of *p*_0_ and *p*_1_ by setting the transmission rate *τ*, while we set the rotation angle *θ* by inserting two half-wave plates into the beam (see Fig. 1). We set in the experiment *τ* and *θ* in a way that Bob receives states which can be expressed as


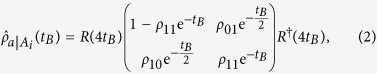


where 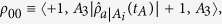 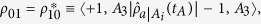 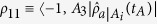

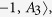
 and 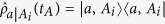. The time *t*_*B*_, between Alice’s and Bob’s measurements, is measured in units of the inverse of the damping constant *γ*. In our experiment, we set *γ* = 1/*t*, where *t* = 50 ns is the time needed for a photon to make one loop in the setup. The time *t*_*B*_ = −*t* log *τ* is set by changing the value of *τ*. The states in Eq. (2) correspond (up to a unitary rotation) to the solution of a simple relaxation^44^ model that provides an example of nonunitary dynamics.

Finally, Bob performs polarisation analysis with a setup consisting of a set of a half- and quarter-wave plates, a polarising beam splitter (PBS), and a single-photon counting module (SPCM). This allows him to project the incoming photons on each of the |*b*, *B*〉 states. Bob receives photons arriving from both channels *λ*. As a result of Bob performing his projections on each of the six states |*b*, *B*_*j*_〉, we obtain the probability distribution *P*(*a*, *b*|*A*_*i*_, *B*_*j*_) (we know what state has been sent by Alice) that holds the same information as the assemblage 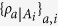. This is because 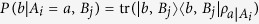. However, in the context of the QKD protocols we are interested only in the compatible bases (*i* = *j*), therefore, 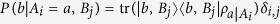, where *δ*_*i*,*j*_ is the Kronecker delta.

**Figure 2:**
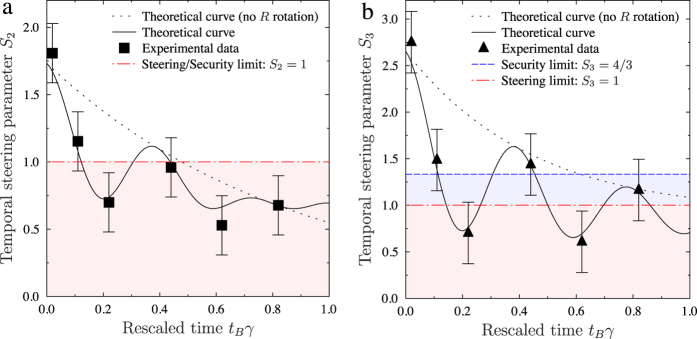
Evolution of the TS parameters *S*_*N*_ corresponding to the TS inequality^21^,^33^: (**a**) *S*_2_ for BB84 (implemented with the eigenvalues of the Pauli operators *σ*_1_ and *σ*_2_) and (**b**) *S*_3_ for B98. Here, *γ* is the damping constant and *t*_*B*_ is the time of the nonunitary evolution between the measurements of Alice and Bob leading to the state defined in Eq. (2). The values of *S*_3_ and *S*_2_ if the rotation *R*(4*t*_*B*_) is not implemented (noise for *σ*_1_ and *σ*_2_ measurements is uniform) are given by the dotted curves 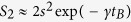 and 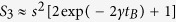, respectively. This also corresponds to our experiment for 4*t*_*B*_ = 2*nπ*, where *n* = 0, 1, 2. The shrinking factor *s* = 0.96 takes into account the initial impurity of the states sent by Alice. The solid curves correspond to *S*_2_ and *S*_3_ calculated for the state given by Eq. (2) and accounting for the setup imperfections described in the Methods.

**Figure 3:**
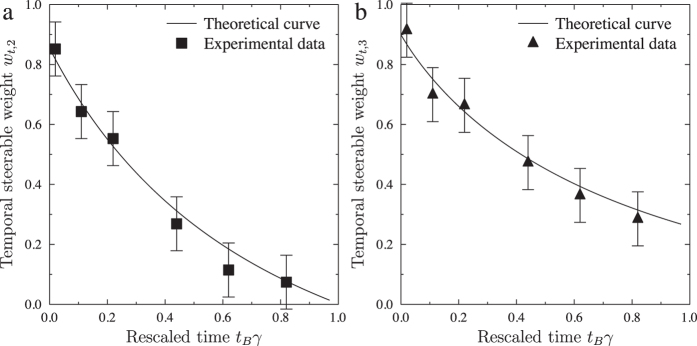
Evolution of the temporal steerable weights *w*_*t*,*N*_ as defined in eq: *sw_def_*. Here, *t*_*B*_ is the nonunitary-evolution time, *γ* is the damping constant, and *N* stands for the number of MUB, calculated for the theoretical (curves) and experimental assemblages (data points) with a semi-definite program for (**a**) BB84 (*N* = 2) and (**b**) B98 (*N* = 3). The solid curves correspond to *w*_*t*,2_ and *w*_*t*,3_ calculated for the state given by Eq. (2) and accounting for the setup imperfections described in the Methods. The temporal steerable weights do not exhibit an oscillatory behaviour because these do not depend on the choice of Bob’s measurement bases.

**Figure 4:**
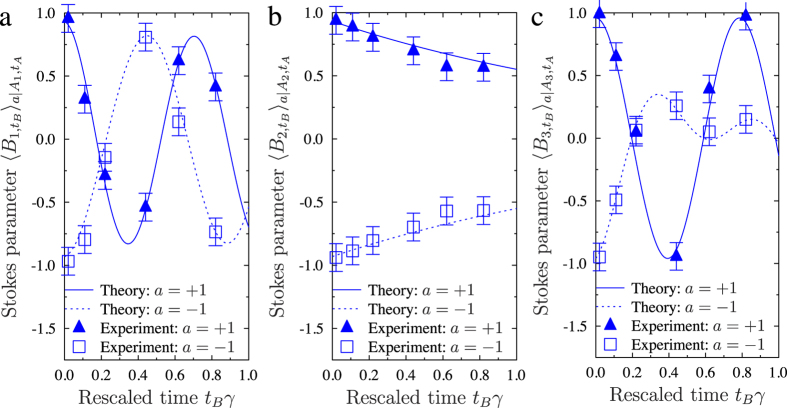
Evolution of the generalised Stokes parameters, i.e., the expected values of the Pauli operators *B*_*n*_ = *σ*_*n*_ (see ref. 33) for the six eigenstates |*a*, *A*_*n*_〉 for *n* = 1, 2, 3 and *a* = ±1. Experimental data for the relevant states with *a* = +1 (*a* = −1) are illustrated with triangles (squares). The time between Alice’s and Bob’s measurements *t*_*B*_ is given in units of the inverse of the relaxation constant *γ*. In our experiment *γ* = 2 × 10^7^ s^−1^. The solid (dashed) curves correspond to the expected values of the Pauli operators calculated for the relevant *a* = +1 (*a* = −1) states defined in Eq. (2).

Figures 2, 3 and 4 show that our experimental data are in good agreement with the expected results. However, the correspondence is not perfect due to experimental imperfections. From these measured results we calculated the TS parameters *S*_*N*_^21^,^33^ and the corresponding TS weights *w*_*t*,*N*_^33^,^39^. The TS inequality [see Fig. 2] provides a sufficient condition for the existence of TS and a security threshold for the MUB protocols with symmetric noise against individual attacks. In our experiment this threshold for *S*_*N*_ is usable only for BB84 (*N* = 2) at a specific time *t*_*B*_ = *nπ*/2, where *n* = 0, 1, 2. In these cases, BB84 is secure against individual attacks if the TS inequality is violated, i.e., *S*_2_ > 1. However, it is not so for B98, where the protocol can be insecure even if *S*_3_ > 1. In B98, we deal with the asymmetric dynamics of the channel (i.e., a relaxation process to one of the eigenstates of the Pauli operators); however, we can assess the security using the *S*_3_ > 4/3 condition. The increase for *t*_*B*_*γ* > 0.8 of the *S*_2_ parameter with respect to the theoretical curve, excluding the *R*(4*t*_*B*_) rotation around the *y* direction, is caused by interchanging the noise between the *z* and *x* directions. The TS weight, as shown in Fig. 3, which is insensitive to rotations, proves or disproves the existence of TS. Comparing Figs 2 and 3, it is clear that the relation between the TS inequality and the TS weight is not trivial. The TS weight implicitly includes all possible TS inequalities, so it detects steering better than the TS inequality. In Fig. 3, the value *w*_*t*,*N*_ = 0, implies the insecurity of the relevant QKD protocol.

## Discussion

We note that a well-known technique for analysing the security in QKD is to introduce virtual entanglement by conceptually replacing state preparation with measurements on an entangled source^45^. Thus, one could think that the standard steering inequalities applied to the virtual entangled source can be used to determine the security requirements on the preparation and measurement correlations. Nevertheless, the described idea corresponds to analysing the security in QKD via spatial or spatio-temporal steering. In contrast with this idea, we analysed the security in QKD via purely temporal steering by replacing the two-qubit measurements with measurements on a single qubit, followed by the evolution under some noisy quantum channel.

In optical fibres one deals with several types of noise and losses which limit the range of the applicability of QKD. These problems are the polarization-dependent losses, geometric phase, birefringence, and polarization mode dispersion (see ref. 36). In our experiment, we implemented a combination of the polarization-dependent losses and polarization rotation [see Eq. (2)]. The polarization-dependent losses can be significant in components like phase modulators and open a way for attacking QKD protocols, e.g., the two-state protocol^46^ by changing nonorthogonal states into orthogonal ones^47^. However, the state-dependent losses are usually not that important in optical fibres. The polarization rotation could be attributed to the geometric phase (a special case of the Berry phase^48^) that accumulates, e.g., when polarised photons are transmitted through fibre loops. Alternatively, this polarization rotation can be caused by polarization-dependent dispersion due to the stress applied to optical fibres. The latter effect occurs in the polarization controllers used in our setup shown in Fig. 1.

Our analysis remains unchanged after including such additional state-independent losses like lossy quantum channels or imperfect detectors. State-dependent losses may cause some basis states to be transmitted more often than others. Thus, the security threshold should be revised by replacing the previously used *r*_*N*_ with its optimal value found for the new asymmetric qubit distribution. This value can be calculated efficiently by optimising the the average single-copy fidelity *F* = 1 − *r*_*N*_ of 1 → 2 qubit cloners^49^.

## Conclusion

We experimentally demonstrated the possibility of temporal quantum steering with photon polarizations in a linear-optical setup. We applied TS for testing the security of two popular quantum-key distribution protocols (i.e., BB84 and B98), which are based on mutually-unbiased bases. We have measured the evolution of the TS weight^33^,^39^ and TS parameters corresponding to the violations of the TS inequalities of Chen *et al*.^21^. To our knowledge, this is the first experimental determination of the TS weight. Note that this TS weight is closely related to spatial steerable weights^50^,^51^, which have not been measured yet. Our experimental tests demonstrate the monogamy of TS and, thus, the security of the analysed cryptographic protocols against individual attacks. We believe that these first experimental demonstrations of TS can lead to useful applications in secure quantum communication.

## Methods

### Pauli operators in the photon-polarisation basis

We apply the standard eigenstate expansions of the Pauli operators, which read as: 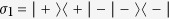, 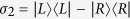, and 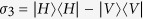, where 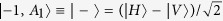, 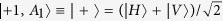, 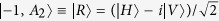, 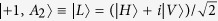, 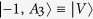, and 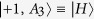. These eigenstates of the Pauli operators are, respectively: the antidiagonal, diagonal, right-circular, left-circular, vertical, and horizontal polarization states.

### Experimental setup

Alice’s setup, as shown in Fig. 1 (in the main article) consists of Q_*A*_ and H_*A*_ that allow her to set any of the |*a*, *A*〉 states. The polarisation modes are flipped *V* ↔ *H* by H_1_, then separated by BD_1_ and *H*-polarised photons are attenuated by the NDF. Next, the polarisation modes are recombined by first flipping back the polarisation modes *V* ↔ *H* by H_2_ and then joining the beams at BD_2_. The channel performs the operation *R*(*θ*) with wave plates H_3_ and H_4_. Each of these two plates implements the transformation that flips the polarisation direction along their optical axes. It can be readily shown that the two transformations constitute a rotation by angle *θ* = 2*δ*, where *δ* denotes the angle between the optical axes of the two wave plates. The polarisation controllers are used to stabilise the output polarisation. To satisfy the consistency conditions (see ref. 33) we assume that all the photons sent by Alice reach Bob, i.e., we interpret all the physical photon losses due to the imperfections of the photon counting process as the result of state preparation and not as the true transmission losses. However, the photons in the state *R*(4*t*_*B*_) |*V*〉, which are lost due to the NDF of transmittance *τ*, are added to the final counts, i.e., the NDF is interpreted as a part of Bob’s detection setup.

Bob’s setup consists of Q_*B*_ and H_*B*_ followed by a PBS and SPCM (Perkin-Elmer). This setup allows to project the incoming photons onto every of the states |*b*, *B*〉. The beam splitter (BS) is used to verify if Alice has indeed prepared photons in the desired state |*a*, *A*〉 before sending them through the channel. However, the purity of states sent by Alice is *p* ≈ 96%, i.e., Bob’s results are effectively scaled by the shrinking factor 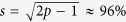. Moreover, the BS rotates the photons travelling to Bob by circa 7° around the *z* axis with respect to the photons travelling to Alice. We take these factors into account in the presented theoretical curves, unless stated otherwise. The time delay, between the photons send via the delay loop and reflected to Bob, directly allows to analyse both the input and output states using the same detection setup. Single photons are generated using a heralded single-photon source. This source uses a type-I spontaneous parametric down-conversion (SPDC) process occurring in a 1 mm thick BBO crystal pumped by the third harmonics (355 nm) of a Nd-YAG laser (300 mW) with a repetition rate of 2500 Hz and a pulse width of 6.5 nm. The signal photon generated in the SPDC process powers the experiment, while the idler is used for triggering. We registered circa 2000 such photon pairs per second. The triggering allows us to post-select only on valid detection events (by eliminating detector dark counts) and to gate the signal detection corresponding to the direct reflection on the beam splitter BS shown in Fig. 1 of the article (no runs in the loop) from one, or possibly more runs, in the fibre loop. Our source delivers signal photons with a polarization-state purity of about 96% and a beam transversal profile corresponding to the TEM_00_ mode filtered by single-mode fibers. The generation rate was adjusted so that the probability of more than one photon impinging on the detector was limited to about 5%.

### Experimental losses

The experimental data collected by Bob are shown in Fig. 4. The setup implements the intended transformations with an average fidelity of circa 95%. We used this value to estimate the sizes of the average error bars presented in Figs 2, 3 and 4. Moreover, the setup dephases the transmitted photons, which results in the attenuation of the off-diagonal density matrix terms by an additional factor of exp(−0.05*t*_*B*_). The setup introduces polarization-dependent losses, which are described by the ratio of the maximum achievable transmissivity for *H*-polarised photons and the maximum transmissivity for *V*-polarised photons (no *V* polarisation filtering) equal to *T*(*H*)/*T*(*V*) = 96%. Finally, there are some technological polarization-independent losses that do not affect our results, leading to a single-loop transmissivity *T*_tech_ = 10%.

### Temporal steerable weight

The temporal steerable weight is a counterpart of the EPR steering weight of Skrzypczyk *et al*.^50^, where the assemblage 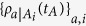 is formed by the set of Alice’s measurements and outcomes. The conditional probability 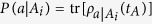 of Alice detecting the outcome *a* while setting her apparatus to measure *A*_*i*_ can be calculated directly from the assemblage. Bob at time *t*_*B*_ receives states 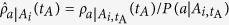 after they passed through a nonunitary channel. Thus, Bob’s assemblage is 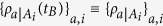, where the explicit form of *ρ*(*t*) is given in the main article. To obtain this assemblage experimentally, Bob performs quantum state tomography of the received qubit.

The unsteerable assemblages^50^ can be created independently of Alice’s observables, and can be expressed as


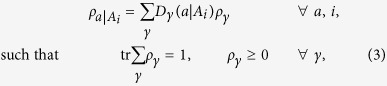


where *γ* is a random variable, *ρ*_*γ*_ are the states received by Bob, and *D*_*γ*_(*a*|*A*_*i*_) are deterministic functions which assign *γ* to a specific measurement *A*_*i*_ and its outcome *a*^33^,^39^,^50^.

The TS weight *w*_*t*_ is the minimal amount of strictly steerable resources needed to split any assemblage as


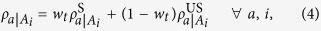


where 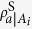 is a steerable assemblage and 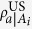 is an unsteerable assemblage. The smallest possible value of 0 ≤ *w*_*t*_ ≤ 1 in Eq. (4) corresponds to the TS weight. Thus, to find its value one needs to solve a convex optimization problem. For small matrices, this can be done efficiently using semi-definite programming.

## Additional Information

**How to cite this article**: Bartkiewicz, K. *et al*. Experimental temporal quantum steering. *Sci. Rep.* **6**, 38076; doi: 10.1038/srep38076 (2016).

**Publisher's note:** Springer Nature remains neutral with regard to jurisdictional claims in published maps and institutional affiliations.
